# Adpositions and presuppositions

**DOI:** 10.1186/s40064-016-2500-2

**Published:** 2016-06-24

**Authors:** Alan Reed Libert

**Affiliations:** University of Newcastle, Callaghan, Australia

**Keywords:** Adpositions, Prepositions, Presuppositions

## Abstract

This paper looks at presuppositions of adpositions, a topic which has not been examined much, in spite of the very large body of work on presuppositions. Some earlier assertions about adpositional presuppositions turn out not to be relevant, because (1) they are incorrect (2) *presuppose* and/or *presupposition* are not used in a technical sense in them, or (3) the presuppositions involved are not unique to adpositions. Some adpositions, e.g. *despite*, have been claimed to be factive, and thus could be presupposition triggers, but it is difficult to determine this, due to the fact that their complements are arguably always themselves presupposition triggers. On the other hand, directional adpositions are clearly presuppositional, as they trigger presuppositions about the location of an object/or entity before or after the motion whose description they are partly responsible for. Such facts may lead one to speculate about word classes and presuppositionality in general, and I will briefly discuss this issue.

## Background

Adpositions are not among the best-known or most discussed presupposition triggers, and there does not seem to be much research specifically on adpositions and presupposition.^1^ In this paper I will investigate to what extent, if at all, adpositions are involved in creating presupositions. It will turn out that many claims about the presuppositionality of adpositions are not correct, if we are thinking of presuppositions in the usual technical sense(s), and that there are few presuppositional adpositions. Thus to some extent this paper will be a review (and criticism) of the relevant literature. However, there are some adpositions which can (or must) trigger presuppositions, including several which have until now escaped attention, to my knowledge, and they will be discussed. We thus find that like verbs, the class of adpositions includes some words that are presupposition triggers (a minority) and many words that are not.

The structure of the rest of this paper is as follows. In “Adpositional ‘presuppositions’ which may not be such or which are not theoretically interesting” I look at and criticize some claims which have been made about presuppositions of adpositions; in my view these claims are either incorrect or not specific to adpositions and hence not of interest if we are focussing on adpositions. In “Factive prepositions” I discuss possible presuppositions of factive prepositions; if there are factive prepositions, one would expect them to be preupposition triggers, like factive verbs. Several adpositions have indeed been asserted to be factive, but it may not be possible to verify these claims, as (according to some views), their complements themselves are always presupposition triggers. In “Directional adpositions” I examine the possibility that directional adpositions can trigger presuppositions, specifically about the location of objects or entities before the motion described by the clause in which they occur. As far as I know, the possible presuppositions of such adpositions have not previously been studied. The final section serves as the conclusion to the paper; in it I will make some more general remarks about presuppositions and word classes.

## Adpositional “presuppositions” which may not be such or which are not theoretically interesting

One must be careful when looking at previous work which apparently discusses presuppositions of adpositions; this is because aside from their technical sense, *presuppose* and *presupposition* have everyday senses which are somewhat different,^2^ and even linguists may sometimes use these words in the latter senses, or they may think that they are using these terms in the standard linguistic senses when they are not. Consider, for example, Feigenbaum’s (2002) paper on prepositions of French and Hebrew, in which these words occur several times, first in relation to the following examples (p. 171), which are given to illustrate the “deletive” meaning of French *sans* ‘without’:

(1a): une maison sans jardin
  ‘a house without a garden’
(1b): une maison avec un jardin
  ‘a house with a garden’

Feigenbaum (ibid.) says:

Deletion can be defined as the relationship between a positive and a negative referent, where the negative referent presupposes a contradictory relationship with its affirmative counterpart. Thus, statement (1a) presupposes that houses have gardens and can therefore be considered to be antonymic with (1b).

First of all (1a) is not a “statement”, as it is simply a noun phrase. Further, it is not the kind of noun phrase which is frequently mentioned in lists of presupposition triggers (e.g. Levinson 1983:181–184), as it is indefinite; compare *the garden behind John’s house* (as in *The garden behind John’s house is/isn’t large*), which presupposes the existence of the garden in question. I think that (1a) involves a conversational implicature rather than a presupposition^3^: if houses did not commonly have gardens, it would be unnecessarily prolix to speak of a house without a garden, as it would be to order a hamburger without celery, since hamburgers are not generally served with celery. (I myself when ordering hamburgers often ask for them to be served without catsup, which is a sensible thing to do, since, at least in some eating establishments, the default situation is for them to have catsup.)

Consider also the following passage from Svenonius (2007:76):

S-selection is semantic selection, and is usually understood to hold of all the arguments of a head, not just its complements …. In this context I am interested in the s-selection by P for its complement; s-selection frequently surfaces in the form of presuppositions. For example, *in* presupposes that its complement be a container, and is infelicitous when the complement is not container-like. Being a presupposition, the requirement is preserved under negation (#*The cat sat in the mat* is odd in the same way as #*The cat didn’t sit in the mat*). Similarly, *among* takes a complement which is complex, *between* takes a complement which consists of two parts, *inside* takes a complement which has ‘sides,’ and so on

The reference to “constancy under negation” (Levinson 1983:168) shows that Svenonius is using *presuppose* and *presupposition* in a technical sense,^4^ or believes that he is, and, more specifically, in a semantic sense (as opposed to a pragmatic sense).^5^

 However, the “presuppositions” involved are rather different from the examples usually brought up in discussions of semantic presupposition, e.g. the “King of France” and “stopped smoking” types of examples. Could they, however, fit under a pragmatic view of presupposition, such as that expressed by Stalnaker (1973:447)?

A person’s presuppositions are the propositions whose truth he takes for granted, often unconsciously, in a conversation, an inquiry, or a deliberation. They are the background assumptions that may be used without being spoken - sometimes without being noticed

It seems that they could, but aside from being different from “conventional or semantic” (Beaver and Geurts 2014) presuppositions, they are also rather different from “conversational” (ibid.) presuppositions e.g. “the presupposition that the interlocutor speaks English” (ibid).^6^ One could say that Svenonius’ putative presuppositions of *in*, etc. are metalinguistic presuppositions (or assumptions), at least as he expresses them, because they have to do with properties of linguistic items (namely what their complements can be),^7^ though of course these properties are connected with properties of the real world. If our notion of presupposition covers these metalinguistic assumptions, then the door is open for various other phenomena to be seen as presuppositions, e.g. c-selection and conventional implicatures.

Magidor (2013:141) has the same general idea as Svenonius;^8^ in order to explain “category mistakes” such as that in (2):

(2): Jill ducked under the theory of relativity. (ibid.:140)

she says, “the infelicity of all these category mistakes can be explained as instance of presupposition failure”; the presupposition in this example is triggered by *under*. Later (p. 145) she states:

the infelicity of [(2)] can be explained by assuming that ‘x φ-ed under y’ triggers (roughly) the presupposition that y is located in space (or, assuming prepositions are generally of type <e, <<e,t>, <e,t≫>, the lexical entry for ‘under’ would be <λy.λφ.λx.x φs under y, y has spatial location>); Since in standard contexts speakers take it for granted that … [the] theory of relativity has no spatial location … and [(2)] suffer[s] from presupposition failure and [is] thus infelicitous.

More generally, “Atomic category mistakes are accounted for by the claim that a wide range of expressions (including most verbs, adjectives, adverbs, and prepositions) are presupposition triggers” (ibid.) (Note that she does not refer to Svenonius in this book, indicating that she was unaware that he had put forth a similar idea.) However, Magidor says (p. 1) that “The king of the United States is drinking water” is a different kind of error than the category errors that she cites there (e.g.”John is drinking the theory of relativity”), and this seems odd, because according to her, both involve presupposition failure—are there then different kinds of presupposition or different kinds of presupposition failure? It would appear so, as she says (p. 146), “Having argued that category mistakes are infelicitous because they suffer from presupposition failure, a natural question one might raise at this point is what separates category mistakes from other instances of presupposition failure?” If one really believed that category errors were presupposition failures, the answer to this question would be “nothing”; one would not posit any significant differences between e.g. presuppositions triggered by a definite description and those triggered by a factive verb (other than obvious superficial differences).

However, in Magidor’s favor is the fact that the “presuppositions” which she mentions seem to pass the projection tests (pp. 134–138). The first projection test is simply the constancy under negation test, but, in my view, constancy under negation is not a sufficient test for presupposition.^9^ For one thing, c-selection and conventional implicatures survive negation, e.g. in both *Mary is poor but honest* and *It is not true that Mary is poor but honest* the presence of *but* leads one to conclude that the speaker thinks that there is a contrast between being poor and being honest. Similar remarks apply to conditionals. Her supposed presuppositions also pass the projection tests involving conjunctions and questions, but once again, so will various other types of things, such as conventional implicatures.

Even if Magidor is correct, the presuppositions of adpositions which she posits are not of a type particular to this word class, and so are not of particular interest if we are focussing on presuppositions of adpositions.^10^

On the other hand, Tyler and Evans (2003) bring up supposed presuppositions which may be restricted to adpositions and perhaps words of other classes with meanings similar to those of adpositions. In their paper on the polysemy of *over*, they state (p. 49), “a preposition presupposes a TR and LM, which are typically supplied linguistically, e.g. *The picture* [TR] is *over the mantel* [LM]”. *Trajector* (TR) and *landmark* (LM) name concepts of Cognitive Linguistics, the framework which Tyler and Evans are using; they (p. 110) describe these concepts as follows:

We call this abstracted mental representation of the primary sense [of a locative preposition] the *protoscene*. It consists of a schematic trajector (TR), which is the locand (the element located, and in focus), and it is typically smaller and movable; a schematic landmark (LM), which is the locator (the element with respect to which the TR is located, and in background), and is typically larger and immovable, and a conceptual configurational-functional relation which mediates the TR and the LM.

Although the “presupposing” which Tyler and Evans speak of does pass the negation test (*The picture is not over the mantel* also leads one to think that there is a picture and a mantel (or, more generally, that there is a “locand” and a “locator”)), they do not seem to be using *presuppose* in the technical sense.^11^ Andrea Tyler (p.c.) has verified this.^12^

However, their sentence does bring up a pitfall one might encounter when looking for presuppositions of adpositions: *The picture is over the mantel* certainly does presuppose the existence of a picture and a mantel, but this is because of the definite NPs *The picture* and *the mantel*, definite NPs being among the most familiar presupposition triggers, and not because of the preposition *over*. Given that most adpositional objects are NPs, and that even some indefinite NPs have been argued to be presupposition triggers (e.g. by von Fintel 1998), athough not specifically those in the complement position of PPs, a large proportion of sentences containing adpositions will have presuppositions about the existence of entities or objects named by their objects, but these presuppositions will not be of interest to us as they are not triggered by the adpositions themselves. Most probably, we will have to disregard many or all existential presuppositions in such contexts (though adpositions may be triggers of other types of presupposition); adpositions do not seem to be able to trigger existential presuppositions, which should not be a surprise—some verbs can trigger presuppositions, but not those of the existential type.

In their (2001) book on the Tungusic language Udihe, Nikolaeva and Tolskaya say the following when discussing postpositions of this language which mean ‘with’ in the comitative sense:

The postposition *mule* ‘with, as distinct from *geje* and *zuŋe*, presupposes a close “inalienable” association between two participants, which must constitute a “natural” pair: a husband and wife, a mother and son, cf.: *ogbö eni mule* ‘elk with female’. So (772a) below can only be understood as meaning that I climbed the tree with my own (and not somebody else’s) younger brother.

Their example (772a) is below:

(3): Bi neŋu mule mo:-tigi tukti-e-mi
  me younger.sibling with tree-lat^13^ climb-past-1sg
  ‘I climbed the tree with my younger brother.’ (ibid.:413)

Once again the “presupposition” probably passes the negation test, i.e. I assume that the negative version of (3) also gives the impression that *neŋu* refers to the younger sibling of the speaker. However, also once again, I am dubious about whether this is presupposition in the relevant sense; if *neŋu* does not refer to the younger sibling of the speaker, (3) will be semantically ill-formed but not in the same way as “The present King of France is bald” is—I think that it would still have a truth value, unlike the King of France sentence (on a semantic rather than pragmatic treatment of presupposition). Irina Nikolaeva (p.c.) and Maria Tolskaya (p.c.) have confirmed that in this paper *presupposes* was not meant in a technical sense; the former author said, “It was meant in a general sense: “requires a close inalienable association”.^14^

Kemmer and Bat-Zeev Shyldkrot (1996:366) discuss the difference between French *toucher* and *toucher à*, both meaning ‘to touch’, as in the following examples (ibid.):

(4a): Cet enfant touche à tout ce qu’il voit.
  ‘That child touches everything that he sees.’
(4b): Elle a touché le radiateur.
  ‘She touched the radiator.’

They say:

there is a subtle distinction in usage between these forms: a greater degree of intentionality is associated with *toucher à*, as in the case of [(4a)], in which the child is touching objects on purpose, presumably to explore them. *Toucher* without the preposition presupposes nothing about intentionality, and is the normal form to use in situations where the subject participant has accidently touched something.

If *toucher* alone does not have a presupposition “about intentionality”, we can infer from the above passage that *toucher à*, and specifically *à* in this context, does have such a presupposition. However, it is not clear to me that it is a presupposition, even though it seems to survive under negation: in one reading *Cet enfant ne touche pas à tout ce qu’il voit* the child touches some things, but not everything that he sees, and this touching was done intentionally. One might argue that this is not a presupposition, but rather it is an entailment. Consider an English sentence with the same meaning:

(5): That child is intentionally touching everything that he sees.

This sentence of course gives the impression, and in fact entails, that the touching was intentional. If we negate it, forming *That child is not intentionally touching everything that he sees*, and if we put stress on *everything*, there is still the impression that the touching is intentional, but I would say that this is because the word *intentionally* entails (and in fact explicitly expresses) intentionality. This means that here, and more generally, as I have already remarked, constancy under negation may not always be a good test for presupposition.^15^

We have seen that many uses of “presuppose(s)” and “presupposition(s)” in connection with adpositions do not seem to involve presupposition in the technical sense, and that some uses of these terms in a technical sense may not be correct or may involve “presuppositions” which seem rather different from presuppositions as they are generally conceived of. Further, some of the presuppositions which have been attributed to adpositions are not limited to adpositions (those involving s-selection), and thus are not of interest to a study specifically on adpositional presuppositions. We now turn to some adpositions which may trigger presuppositions.

## Factive prepositions

Factive verbs are often listed among presupposition triggers. If there are factive words belonging to other parts of speech, presumably they will also trigger presuppositions of the same type. Although verbs are the words most commonly associated with factivity, there are also (according to some authors) factive nouns (see e.g. Vendler 1980:280), adjectives (see e.g. Norrick 1978), adverbs (see e.g. Geuder 2000:111), conjunctions (see e.g. Hengeveld 1998:354–355), and pronouns (see Cornish 2015).^16^ The only main part of speech of which I have not seen any members asserted to be factive (in the relevant sense) is the interjections.^17^ I also have not found any references to factive numerals. It would therefore not be a surprise to see references to factive adpositions, but there seem to be very few such references (in the relevant sense of *factive*).

Van Dijk (2011:45) does cite *despite* as a factive preposition.^18^ However, he does not discuss it in detail. It is difficult to test it for presuppositionality, since its objects will often or usually trigger presuppositions by themselves. Consider for example the following sentences:

(6a): Despite their opposition we continued with the project.
(6b): Their opposition caused much unhappiness.

Among the presuppositions of (6a) is something like ‘they were opposed to a project’, but this is also a presupposition of (6b), which lacks prepositions. We therefore need sentences without adpositional objects, e.g. definite NPs, which are presupposition triggers.

It may not be sufficient to place indefinite NPs in the complement position of adpositions, since, as mentioned above (according to some authors) they, or at least some of them, can also trigger presuppositions, as in the following examples:

(7a): Despite an increase in opposition we continued with the project.
(7b): An increase in opposition caused us to drop the project.

Both of these examples presuppose that there was an increase in opposition (among some people) for the project in question; since *despite* does not occur in (7b) it cannot be responsible for the presupposition (that there was an increase in opposition) which these examples share.^19^

One might think that gerund constructions can be adpositional complements which may not by themselves trigger presuppositions (or at least not the same kind of presuppositions), as in the examples below:

(8a): Despite opposing us for weeks, in the end he helped us with the project.
(8b): Despite his opposing us for weeks, in the end he helped us with the project.
(8c): Despite him opposing us for weeks, in the end he helped us with the project.

Here we have what one could see as a factive presupposition (‘he opposed us for months’). However, one might argue that this is not due to the presence of *despite*, since in the absence of this word there the same presupposition exists.

(9): Opposing us for weeks, he finally gave in and helped us with the project

I may not find this sentence to be perfectly well-formed, but similar sentences are clearly well-formed, e.g. *Having missed my bus to the airport, I had to take a taxi*. Such sentences, specifically the gerund construction part, carry the same sort of factive presupposition, e.g. (9) presupposes that he (whoever he was) opposed us for weeks. The literature contains other examples of gerund constructions which are said to have presuppositions, e.g.:

(10a): Fred allowed Mary’s sleeping in on Saturdays. (Asher 1993:191)
(10b): John’s hitting Mary was a bad thing to do. (ibid.)
(10c): Him hitting her so hard frightened Mary. (ibid.:194)
(10d): John sprinting past Bill was a welcome sight. (ibid.:196)

The first two examples have “POSS-*ing* constructions” (Asher 1993:190). Asher (1993:191) says, “[(10a)] has a factive presupposition or at least implicature … The presupposition is that Mary did sleep in on Saturdays. [(10c)] carries a similar presupposition”. One might not be surprised that they cause presuppositions, since possessed NPs in general trigger presuppositions, e.g. *John’s guitar* triggers the presupposition ‘John has a guitar’. This could be attributed to the fact that they are definite NPs. While (10a-b) do not involve possession in a literal sense, the gerund constructions in them are still definite, e.g. there might be various acts of hitting, or of hitting Mary, but *John’s hitting Mary* refers to one particular act. *Mary’s sleeping in on Saturdays* does not refer to one particular event, but it does refer to a particular series or set of events.^20^

(10c) and (10d) involve an “ACC-*ing* construction” (ibid.:196), about which Asher (ibid.) says the following:

ACC-*ing* constructions introduce a possibility discourse reference and a factive presupposition. Some predicates of ACC-*ing* constructions may block this factive construction, in which case the the ACC-*ing* construction will denote a possibility. If nothing blocks the factive presupposition, the ACC-*ing* construction will denote a fact.

More generally, Ormazabal (2005:105) gives “gerundival constructions” as an example of “complement[s] that cannot be non-factive”, as shown by the following (ibid.) examples:

(11a): *John thought/believed [the fact that the earth is round]
(11b): *John thought/believed [Mary going to the movies]

Presumably (11a) is ill-formed because a factive complement (*the fact that* …) is the complement of a non-factive verb; if (11b) is ill-formed for the same reason, then *Mary going to the movies*, and other gerund constructions, are necessarily factive. There are clearly some sentences containing gerund constructions which do not have factive presuppositions, e.g. *Going to the movieswould be nice*. Asher might argue that in such cases the factivity of *Going to the movie*s is cancelled, as with the example in note 20; in the present case the modality of the main clause would cause the cancellation.^21^

Portner (1995:620) gives the following examples of sentences containing gerunds:

(12a): It’s unlikely that calling the fire station brought help.
(12b): Lifting those clocks didn’t tire me out.
(12c): Sam didn’t believe that planting cacti tired me out.
(12d): If planting cacti tired Sam out, he will surely die.

He states (ibid.), “the events picked out by the gerunds [in these examples] are presupposed to have occurred”. Notice that these examples are neither POSS-*ing* constructions nor ACC-*ing* constructions, as they do not have an overt subject. Thus all of the major types of gerund construction have been said to trigger presuppositions. However, Portner does not think that all types of gerund construction are presuppositional; on p. 637 he gives the following examples:

(13a): John imagined Bill’s leaving.
(13b): John imagined/predicted the earthquake.
(14a): John imagined Bill leaving.
(14b): John imagined/predicted an earthquake.

For Portner some gerunds may well be definite and others indefinite, and this property (not surprisingly) is connected with presuppositionality; he says the following (ibid.) about the above examples:

[(13b)] presupposes that a particular earthquake is somehow salient in the conversation, just as [(13a)] presupposes that a leaving is under discussion Neither [(14a)] nor [(14b)] presuppose any event. The difference between [(13b)] and [(14b)] is simply definiteness, and it’s seems [sic] likely that it’s the same difference in [(13a)]–[(14a)].

We can imagine that Asher, or someone with similar views, might say that in fact the gerund construction in (14b) is presuppositional, but this presuppositionality is cancelled by the verb *imagined*. However, there is a complication here: in the passage quoted above the presupposition is actually not a factive one, i.e. Portner is not saying that (13b) involves a factive presupposition (which would be that Bill left), but only a presupposition that an event of leaving is “under discussion” (and it would be hard to imagine it not being under discussion, since it is mentioned in the sentence). Most authors would say that (13a) involves an existential presupposition (that there was an earthquake), not (only) a presupposition (if it is such) that “a particular earthquake is somehow salient in the conversation”. It would appear that Portner has made some kind of error; we might rather say that (13b) presupposes the existence of a particular earthquake [unlike (14a)], and that (13a) has a factive presupposition (that Bill left) [unlike (14a)].^22^

There are thus various views about the factive status of (some types of) gerund constructions, which makes it difficult to determine the factivity of adpositions which take such constructions as complements. If all types of gerund construction are presuppositional, it would be very difficult or impossible to test whether *despite* is a presupposition trigger, as all the complements which it could have (e.g. NPs headed by verbal nouns or by gerunds) are presupposition triggers themselves.^23^ The same applies to other prepositions which can take gerund constructions as complements; (15a) and (15b) create the presupposition ‘he opposed us’ but the gerund constructions alone (without *becauseof*/*due to*) have this presupposition.^24^

(15a): Because of him opposing us, we dropped the project.
(15b): Due to him opposing us, we dropped the project.

If, on the other hand, at least some gerund constructions are not factive (and in my view the best candidates for non-factivity might be gerund constructions without anything overt corresponding to subjects in finite clauses, e.g. *Going to the movies* in *Going to the movies would be nice*), then *despite* and other prepositions which can take gerund complements could be shown to be factive, as in *Despite going to the movies this afternoon, I was still in a gloomy mood*.

If we now look at some other claims about adpositional presuppositionality, Bonyadi and Samuel (2011:9) are incorrect when they state that *given* (as a preposition) causes presuppositions in examples such as the following (ibid.)^25^:

(16): Given Russia’s oil wealth and nuclear arsenal, the West’s leverage is limited, but not inconsequential.

That is, the definite NP *Russia’s oil wealth and nuclear arsenal* alone triggers the presupposition that ‘Russia has oil wealth and a nuclear arsenal’; we cannot test whether *given* adds any presupposition(s) to the sentence.^26^ The presupposition here is existential, but *given* could take a gerund complement, e.g. *Given John’s constant bragging, we expected more from him*, and such cases would involve factive presuppositions. In these cases, as well as in (16), the factivity may be due to the complement and not (only) to *given*, and thus *given* may not be factive.

Bonyadi and Samuel may be also be incorrect about *instead of*, as in the example (ibid.:9) below, being a presupposition trigger:

(17): Instead of defending Zimbabwe’s people and their right to democratic change, he [South African’s president] has shamefully chosen to protect Mr. Mugabe.

That is, if *defending Zimababwe’s people* … is factive, then we need not attribute factivity to *instead of*, or we could say that it is factive, but this factivity is vacuous since its complement is factive.^27^

However, this example is interesting, because my sense of factivity here seems weaker, that is, the presupposition (‘South African’s president did not defend Zimbabwe’s people and their right to democratic change’ (ibid.)) does not seem as strong as presuppositions triggered by *despite*. This may be because I can imagine a type of situation where *instead of* does not trigger a presupposition: if we are discussing a trip to be taken by you and I say:

(18): Instead of going to Philadelphia, you should go to/you should consider going to New York.

Here there is no presupposition that you have gone to or will go to Philadelphia; perhaps this is due to the modality of the main clause, marked by *should*; that is, the main clause, and the whole sentence, is marked as being hypothetical, and thus some presuppositions involved with it may be weakened or may disappear. In (18) there is perhaps a presupposition that the addressee is considering going to Philadelphia (or that this idea is “under discussion”, as Portner would say). However, it could be argued that this is a conversational implicature; it would not be relevant (and thus not felicitous) to utter (18) in a context where the addressee has no plans to go to Philadelphia.

In contrast, it appears that any presuppositions (or implicatures) triggered by *despite* are not affected by modality, as shown below:

(19): Despite being tired, you would be better off if you finished your paper today.

That is, even though this is a hypothetical situation, there is an implication that the addressee is or will be tired.

## Directional adpositions

Let us now turn to another type of adposition. O’Keefe (1996:305) discusses the following sentence:

(20): The icicle fell from the roof to the garden.

On the next page is a drawing meant to show part of the meaning of this sentence, and on p. 305 he says, “The left side of the representation shows the unstated presupposition that the icicle was on the roof for some unstated time prior to the event of the sentence”. I do not think that he is using *presupposition* in its technical sense, because in this sense presuppositions are always unstated. However, there is an insight here, and one might argue that in fact this is a presupposition in the technical sense, and, more generally, that sentences containing *from X* presuppose that the object or entity in motion was at/in/on X before the motion. This seems to be a presupposition rather than (only) an entailment; as indicated by the fact that it passes the negation test (*The icicle did not fall from the roof* also leads one to conclude that the icicle was on the roof), is maintained in questions (*Did the icicle fall from the roof?*), and can be cancelled:

(21): The icicle did not fall from the roof, because in fact it never was on the roof.

To my knowledge, this insight has not been stated elsewhere in the literature on presupposition and *from* has not been listed among presupposition triggers.^28^ It is parallel to *begin to* (whose presuppositions have been discussed in some of the literature) in the sense that an initial, pre-event status is presupposed—a location in the case of *fell from* and a state of not doing something in the case of *begin to*. Note the asymmetry between *from* and *to* in this context, which might appear odd since they are both prepositions, but this asymmetry is parallel to that relating to *begin to*—*begin to* presupposes an initial state of not doing something, but entails the subsequent state of doing that thing, e.g. *John began to run* presupposes that he was not already running (cf. *John did not begin to run because he was already running*), but entails that he was subsequently running; note the semantic ill-formedness of #*John began to run but did not actually run*.^29^ We find a similar situation with *to stop x*-*ing* (a construction which is much discussed in the literature on presuppositions); *John stopped smoking* presupposes that he smoked, but entails that he ends up not smoking, and here the intuitions may be clearer: *#John stopped smoking, but he continued to smoke*.

Similar facts obtain with *leave*, which need not be followed by *from* (but can be); *John left (from) Sydney* presupposes that he was in Sydney, and one might therefore argue that *from* itself is not a preposition trigger (i.e. that *leave* is the trigger of the preposition). However, *from* is required with most other verbs of motion (e.g. *The icicle fell roof to the garden), and so is the only candidate for the trigger of the presupposition with such verbs.

I have said that the preposition *to* entails rather than presupposes being at the destination in sentences such as (20). However *to* does trigger a presupposition, and again there is a parallel with *begin to*: (20) presupposes that the icicle was not (already) on the roof, just as *begin to x* presupposes that the subject was not already doing x.^30^ This presupposition passes the negation test: *John did not go to Sydney* presupposes that *John was not in Sydney*, but one can cancel this presupposition (as one would expect):

(22): No, John did not go to Sydney yesterday, because he was already there.

The situation is the same with the prepositions *into* and *onto*, as well as some other prepositions when used in a directional (rather than a locational) sense, e.g. *under*; *John went under the house* presupposes that he was not already under the house.

Such facts seem obvious, but they have rarely if ever been noted before. It thus seems that there are some clear instances of adpositions acting as presupposition triggers.

## Conclusion

We have seen that many of the non-temporal adpositions which have been asserted to be presupposition triggers turn out not to be so, or the type of presupposition (those involving “category mistakes” or s-selection) which they trigger are not unique to adpositions. However, there may be several factive prepositions (e.g. *despite*), and directional adpositions in general (including *from*, when marking the starting point of motion) trigger presuppositions. The class of adpositions is thus like the class of verbs; both have some members which are presuppositional and some (perhaps the majority) which are not. One reason for this may be that both verbs and adpositions can be involved in the description of motion/direction.

If we think about word classes in general, there are three possibilities for presuppositionality: (1) no members can be presuppposition triggers, (2) some, but not all, members can be presupposition triggers, and (3) all members can be presupposition triggers. As we have seen, the second possibility holds of both verbs and adpositions. As far as I know, there no word classes all of whose members are presuppositional, and in fact it might be difficult to imagine such a language with such a word class, especially the class of verbs; presumably every language must be able to express ‘to know’ and ‘to think’ (or something similar), and there is something in the nature of the states themselves (knowing and thinking) which makes them factive, and hence presuppositional (in the case of ‘to know’) and non-factive (‘in the case of think’)—a factive version of ‘to know’ seems to be the same or similar in meaing to ‘to think’ or ‘to believe’. It is easier to imagine a language all of whose adpositions are presuppositional since there are some languages with very small inventories of adpositions, and so there could be a language with only two adpositions, both of which are directional, and hence presuppositional. However, I do not know of any such language.

While word classes which are completely presuppositional may be rare or non-existent, as I noted above, there are (at least) two word classes which have no presuppositional members, interjections and numerals. While numerals can create conversational implicatures (of the scalar type), it is hard to imagine (at least for me) how a number could trigger a presupposition. It is almost as difficult to conceive of a presuppositional interjection; one possibility might be a factive interjection. For example, if I utter the *Oh!* of surprise, I could either be reacting to an event or situation which I know to be actually happening, or to a possible, but not clearly true, event or situation, and some language could have different words meaning ‘Oh!’ for these two situations. If someone heard the first *Oh!*, they would conclude that whatever surprised the speaker was something the speaker knew to be true. However, this seems far-fetched, and I know of no language with such interjections.

Thus, while there seems to be some connection between word class and presuppositionality, in the sense that some word classes rule out the possibility of being presuppositional, there is not a strong connection, as some or all of the other words classes (e.g. adpositions) have both presuppositional and non-presuppositional members. Whatever property or properties make a word presuppositional seem largely independent of the properties which are responsible for a word being placed in one or another word class.

## Footnotes

1 I will not discuss the presuppositions of temporal adpositions (e.g. *before*), which have received some attention, going back to Frege (1892:42, footnote**). For work on temporal adpositions (or on adpositions which can be used in a temporal sense), see e.g. Gründer (2008), König (1974), and Onea (2011). As noted in Macagno and Capone (2015), the preposition *of* can mean ‘before’ or ‘after’; in such situations it would presumably trigger the same presuppositions as *before* or *after*.

2 See Levinson (1983:168) for discussion and examples.

3 The distinction between presuppositions and conversational implicatures is not always as clear as one might expect (and hope). For that matter, the existence of a separate class of conclusions which we call presuppositions has not been universally accepted, as shown e.g. by the title of Sect. 8 of Atlas (2004), “The Reduction of Presuppositions to Conversational Implicata: Accommodation, Calculability, and Common Ground”.

4 As van der Sandt (1988:37) says, “The oldest and best-known test for determining which syntactic constructions and lexical elements give rise to presuppositions is embedding under negation”. However, he then (ibid.:37–39) mentions problems with this test.

5 Clark (1973) makes assertions about “presuppositions” of various English prepositions which seem at least vaguely similar to those of Svenonius. For example, on p. 40 he says: Consider the *frame A is at/on/in B*. All three prepositions assert that A is in the same location as B, but *at*, *on*, and *in* presuppose that the location of B is a one-, two-, and three-dimensional space, respectively. However, it is not clear whether he (thinks that he) is using *presupposition* in a technical sense.

6 Obviously much depends on how one defines *presupposition*. An anonymous referee has recommended that I define *adposition*. In my view this is not crucial, since most of the adpositions which I discuss are not controversial with respect to which word class they belong to. In Libert (2013) I provide a detailed examination of some words whose status as adpositions is less clear, and I show how difficult the classification of many putative adpoisitions is. For English, Latin, and some other languages, one might define adpositions as words which take no inflection (differentiating them from nouns, pronouns, verbs, adjectives, and adverbs) but which assign case to their complements (differentiating them from conjunctions and interjections). However, this will not work as a universal definition, as there are languages which arguably have inflected adpositions. I will make the somewhat controversial assumption that some sequences of words (e.g. *instead of*) are also adpositions; I am certainly not the only one assuming this, but to accommodate such items, my definition of *adposition* would have to be modified. For one discussion (among many) of defining *adposition* see Lunggren (1951).

7 For another type of metalinguistic presupposition in this sense of *metalinguistic* (‘about language’) see Maier (2014). However, such presuppositions (“quotational presuppositions”, p. 30) seem more like typical presuppositions than the kind brought up by Svenonius. *Metalinguistic preupposition* is also used in another way by e.g. García-Ramírez (2010:151, 153), meaning something like ‘relating to the extralinguistic world’. His (ibid.:153) example is given in (i):

(i): L: Nessie is carnivorous. E: No, she isn’t. She doesn’t even exist.

He says (ibid.) about this example, “In [(i)] L uses ‘Nessie’ referentially. So L’s use carries a metalinguistic presupposition: that ‘Nessie’ has a referent”. I do not mean *metalinguistic* in this sense.

8 Cf. Corblin (2003), who says (p. 3), “Having argued that this typical example of selectional restriction can be described in the same terms than [sic] presuppositions, we are lead to conclude that presupposition might be nothing else than selection”.

9 I am not the only one who holds this view; I have already mentioned (note 5) the fact that van der Sandt (1988:37–39) brings up problems with this test, although the problems which he raises are different from those which I have in mind.

10 Macagno (2015) apparently has the same view of presupposition (i.e. that it includes s-selection), as he (p. 468) gives “*I am walking in the park with Australia” as an example of a presupposition which “can … be triggered by syntactic elements … such as prepositions”.

11 This should not be seen as a criticism of them or of anyone else who uses *presupposition* in a non-technical sense; one might say that it is unfortunate that a term with an everyday meaning was chosen for this sort of implication. This situation does not arise with terms such as *conventional implicature*, which have only a technical sense.

12 She stated: What we were attempting to convey was the idea that most prepositions started out as spatial markers which articulated a spatial relationship between an entity in focus (TR) and a locating/background entity (LM). Conceptually, this relationship between a TR and LM continues. In many uses, prepositions have, of course, changed and extended so that they act as adverbs, etc, so the syntax has changed. However, conceptually, this relationship between a TR and LM continues. On the surface, it may look like the TR-LM relation has disappeared. But even in a sentence like: ‘Class is over’, conceptually there is a TR (the class—a metonymy for the activity of a group of students and a teacher carrying outparticular activities) and the unarticulated LM (analogous to the hur[d]le the horse jumps over)—the period of time the class meets. In order for class to be completed (over), the people involved in the teaching/learning activity must temporally get beyond the allotted time for the activity.

13 lat stands for ‘lative’.

14 Even if *mule* does not trigger a presupposition (in the technical sense), the type of conclusion which it causes (that there is “a close ‘inalienable’ association) is of interest; is it simply part of the (truth-conditional) meaning of the word (I suspect not), or is it some sort of pragmatic conclusion (i.e. using it when there is not a close relationship is inappropriate, but does not cause a proposition to be false)? However, it is beyond the scope of the present paper to investigate this.

15 For discussion of various treatments of presuppositions said to be associated with *à* and another (sometimes) empty French preposition, *de*, see Fraczak (2008). She mentions some situations in which (she claims that) the difference or choice between these prepositions (e.g. *commencer à* vs. *commencer de*, both meaning ‘to begin to’, pp. 178–179) does not depend on the presence or absence of presuppositions connected with them. She states (p. 179), “We can conclude from this part of the analysis that the use of the preposition *de*, as opposed to *à*, cannot be systematically associated with the factor of situational presupposition”. Earlier (p. 174) she says, “The analysis of our corpus of relevant examples leads us to conclude that “lexical” presupposition is present in a large majority of verbs, independently of the preposition used”.

16 Cornish (2015:103) speaks of “a factive pronoun”, e.g. *it* in “Of course anyone who is assaulted can report it to the police” (ibid.); it is also “a bound propositional variable (ibid.) in this sentence. He cites (a 1971 reprint of) Kiparsky and Kiparsky (1970) in which “factive it” (p. 165) is discussed.’ However, this “factive *it*” is not a presupposition trigger (unlike e.g. the verb *know)*, but rather “serves as an optional reduction of *the fact*” (ibid.), and, one might argue, should be called something different (e.g. “factual *it*”), with *factive* being reserved for the meaning ‘triggers a presupposition of the truth of the proposition which it introduces’. It might be difficult to imagine a factive pronoun in this sense, since pronouns do not (generally) have complements or introduce propositions. Capone (2013) does not use the term *factive pronoun(s)*, but he does use the term *presuppositional clitics* (by which he means clitic pronouns (in Italian)); on p. 477 he says, “I called these clitics presuppositional clitics because, at least when there is no context forcing modal subordination, the pronominal clitic serves to promote a proposition to the status of presupposed information (a presupposition)”. One might argue that these items could have been called factive pronouns.

17 On the other hand, not everyone accepts the existence of factive verbs; see e.g. Hazlett (2010), who seems to reject the factivity of words in general.

18 On p. 44 he calls *despite* in the following example (p. 43) a factive adverb: [regrets] that despite sustained diplomatic effort by Her Majesty’s Government it has not proved possible to secure a second Resolution in the UN because one Permanent Member of the Security Council made plain in public its intention to use its veto whatever the circumstances. However, this seems incorrect, as it is clearly a preposition here (and in general).

19 Even when there is no article, a presupposition of this sort can be created:

(ia): Increases in opposition caused us to drop the project.
(ib): Opposition to the project caused us to drop it.

(ia) presupposes that there were increases in opposition, and (ib) presupposes that there was opposition to the project.

20 However, Asher (1993:192) states that “such presuppositions can be cancelled in certain contexts” and gives the example *Mary prevented John’s kicking the cat*.

21 One might then ask why *thought* or *believed* in (11b) cannot cancel the factive presupposition of the gerundive construction; I do not know how people holding views like those of Asher and Ormazabal would answer this.

22 This is not to say that I agree with this view; I am simply stating what Portner might have said if we remove the idea that “salience” or being “under discussion” are presuppositions.

23 An indicatation that such cases involve presupposition (rather than entailment) comes from the negation facts, e.g. both (10d) and (i) presuppose that John sprinted past Bill:

(i): John sprinting past Bill was not a welcome sight.

24 Admittedly, the removal of the presuppositions from these examples makes them less than perfectly well-formed *(??him opposing us, we dropped the project*), but since similar examples are (or have been claimed to be) well-formed, e.g. (10c), it is not unreasonable to think that the ACC-ing constructions in (15a–b) are presupposition triggers (but that they require a preposition for some unrelated reason).

25 They are correct about *given that* being a presupposition trigger, as in (i), but if we consider *given that* to be a conjunction, then it is not relevant for us:

(i): Given that North Korea has made this decision to serve its own national interests, the same approach cannot be used for other countries. (ibid.:10)

.

26 A example from outside the realm of linguistics could help clarify the situation. Let us imagine that some medical condition has been claimed to be fatal, but only to people over the age of 150. We cannot test whether it is in fact fatal, because any possible subjects for such a test would already have died before they reached their 150th birthday (from some other fatal condition or disease). The condition in question may well be fatal to them, but this cannot be proved (or disproved, for that matter).

27 It is curious that there is diagreement about the factivity of some gerund constructions; one might not expect this, since it is fairly clear which verbs are factive (e.g. *know*) and which are not (e.g. *think*).

28 On the other hand, it is not clear to me that O’Keefe is correct when concerning (20) he speaks of (p. 305) “the presupposition that the icicle continues in the garden for some duration after the event”. It is an entailment, not a presupposition, of *to X* that the entity or object in motion reaches (and at least briefly stays) at X. Evidence of this is that it does not survive negation (*John did not go to* Sydney does not lead to the conclusion that he reached or was at Sydney) and cannot be cancelled (*#John went to Sydney but he did not reach it* (only good if *to* means ‘toward’) or *#John went to Sydney but he was never there*).

29 This sentence is actually acceptable with *begin to* meaning ‘prepare to’ or something similar, but this may be a repair reading.

30 Note that the presupposition of *begin to* is not dependant on the infinitival *to*; *begin x-ing* triggers the same kind of presupposition. In both cases the presupposition disappears when *begin (to)* means’ begin to do something (again)’ in a certain period of time, on a sort of iterative reading, e.g. *I worked on the paper until 11 p.m., woke up at 7 a.m. and began to work on it (again)*. One could say that the presupposition is that the subject was not doing x immediately before the beginning of the action, just as *John went to Sydney* does not presuppose that John had never been to Sydney before, but only that he was not there immediately prior to the act of going.
